# 449 A CTS team approach to investigating sEV production in magnetic-responsive scaffolds following Salmonella infection – ERRATUM

**DOI:** 10.1017/cts.2026.10762

**Published:** 2026-07-01

**Authors:** Alexander Schultz, Brian Molina Diaz, Mei He, Mariola J. Ferraro

When this article was originally published in the Journal of Clinical and Translational Science it contained an incorrect order for the list of authors. This has been updated in the original article.

The authors apologise for this error.
